# The *TgsGP* Gene Is Essential for Resistance to Human Serum in *Trypanosoma brucei gambiense*

**DOI:** 10.1371/journal.ppat.1003686

**Published:** 2013-10-03

**Authors:** Paul Capewell, Caroline Clucas, Eric DeJesus, Rudo Kieft, Stephen Hajduk, Nicola Veitch, Pieter C. Steketee, Anneli Cooper, William Weir, Annette MacLeod

**Affiliations:** 1 Wellcome Centre for Molecular Parasitology, College of Medical, Veterinary and Life Sciences, University of Glasgow, Glasgow, United Kingdom; 2 Department of Biochemistry and Molecular Biology, University of Georgia, Athens, Georgia, United States of America; London School of Hygiene and Tropical Medicine, United Kingdom

## Abstract

*Trypanosoma brucei gambiense* causes 97% of all cases of African sleeping sickness, a fatal disease of sub-Saharan Africa. Most species of trypanosome, such as *T. b. brucei*, are unable to infect humans due to the trypanolytic serum protein apolipoprotein-L1 (APOL1) delivered via two trypanosome lytic factors (TLF-1 and TLF-2). Understanding how *T. b. gambiense* overcomes these factors and infects humans is of major importance in the fight against this disease. Previous work indicated that a failure to take up TLF-1 in *T. b. gambiense* contributes to resistance to TLF-1, although another mechanism is required to overcome TLF-2. Here, we have examined a *T. b. gambiense* specific gene, *TgsGP*, which had previously been suggested, but not shown, to be involved in serum resistance. We show that *TgsGP* is essential for resistance to lysis as deletion of *TgsGP* in *T. b. gambiense* renders the parasites sensitive to human serum and recombinant APOL1. Deletion of *TgsGP* in *T. b. gambiense* modified to uptake TLF-1 showed sensitivity to TLF-1, APOL1 and human serum. Reintroducing *TgsGP* into knockout parasite lines restored resistance. We conclude that TgsGP is essential for human serum resistance in *T. b. gambiense*.

## Author Summary

*Trypanosoma brucei gambiense* causes 97% of all cases of African sleeping sickness, a fatal disease of sub-Saharan Africa. Most species of trypanosome, such as *T. b. brucei*, are unable to infect humans due to trypanolytic factors in human serum. Understanding how *T. b. gambiense* overcomes these factors and infects humans is of major importance in the fight against this disease. Previous work indicated that a failure to take up some trypanolytic factors by *T. b. gambiense* contributes to resistance, although other mechanisms are involved. Here, we have examined a *T. b. gambiense* specific gene, *TgsGP*, for involvement in resistance to human serum. We show that *TgsGP* is essential for resistance to lysis as deletion of *TgsGP* in *T. b. gambiense* renders the parasites sensitive to most trypanolytic factors. *TgsGP* deletion in *T. b. gambiense* modified to overcome the sub-species trait to reduce uptake of some trypanolytic factors resulted in sensitivity to all trypanolytic factors. Reintroducing *TgsGP* into these knockout parasite lines restored resistance. We conclude that TgsGP is essential for human serum resistance in *T. b. gambiense*.

## Introduction

Throughout their evolution in sub-Saharan Africa, humans have been under assault by a range of different pathogens. One defining challenge is that posed by African trypanosomes, a species complex of blood-borne protozoan parasites transmitted by tsetse flies [1]. The principle pathogenic species in Africa are *Trypanosoma brucei, T. congolense* and *T. vivax*, although only *Trypanosoma brucei* sub-species are able to infect humans. A key feature of these parasites is the ability to undergo antigenic variation by modifying the variant specific glycoprotein (VSG) enveloping the cell that renders the mammalian adaptive immune system largely ineffective [2]. Components of the innate immune system therefore contribute significantly to defence against these organisms [3]. Critical to these defences is the serum protein apolipoprotein L1 (APOL1) found in some catarrhine primates, including humans [4], [5]. The protein is able to kill the majority of trypanosome species in a dose-dependent manner [5]. APOL1 is delivered to parasites in two fractions of the high-density lipoprotein (HDL) component of serum, termed trypanolytic factor 1 and 2 (TLF-1 and TLF-2) [6]. TLF-1 binds to the parasite through an interaction between the haptoglobin-related protein (HPR) surrounding the TLF-1 particle and the haptoglobin haemoglobin receptor (HpHbR) in the flagellar pocket of the parasite [7]–[9]. Under the acidic conditions found in the lysosome, APOL1 changes conformation and embeds in the lysosomal membrane, forming pores in the organelle, leading to cell death [5], [10]. A proportion of TLF-2 similarly enters trypanosomes via HpHbR, although an alternate route also contributes to uptake [11].

Although TLF-1 and 2 kill the majority of trypanosome species, two sub-species of *T. brucei* have evolved to overcome this innate immunity. *T. b. rhodesiense* and *T. b. gambiense* are both resistant to lysis by APOL1 and establish bloodstream infections in humans [1]. *T. b. rhodesiense* causes an acute form of the disease and is found in East Africa whereas *T. b. gambiense* is found in West and Central Africa. *T. b. gambiense* causes a more chronic form of the disease and is responsible for 97% of all human cases of trypanosomiasis [12]. The mechanism of human serum resistance for *T. b. rhodesiense* involves the expression of a truncated VSG, termed serum resistance associated (SRA) protein [13], [14]. SRA binds to APOL1 in the lysosome, preventing lysis [14]. However, the *SRA* gene is absent from *T. b. gambiense*, the more prevalent human infective sub-species [15]. The *T. b. gambiense* subspecies consists of two sub-groups (1 and 2) that differ in phenotype, including their associated pathology. Group 1 *T. b. gambiense* parasites are the most prevalent of the human infective trypanosomes and are responsible for the vast majority of cases [16]. Group 1 *T. b. gambiense* can be distinguished by both their reduced efficacy of HpHbR for binding TLF-1, due to a conserved single nucleotide polymorphism [17]–[19] and also by the presence of a specific truncated *VSG*, *TgsGP* [20]. The *TgsGP* gene is present in all group 1 isolates examined to date but not in *T. b. brucei, T. b. rhodesiense* or group 2 *T. b. gambiense* [20]–[23]. The specificity of *TgsGP* to group 1 *T. b. gambiense* and its resemblance to *SRA*, in that it is a truncated *VSG* gene, led to a suggestion that this gene may confer human serum resistance to group 1 *T. b. gambiense* [20]. The gene was transfected into *T. b. brucei* where it did not confer increased resistance to human serum. It was hypothesized that if TgsGP was involved in human serum resistance other factors would also be required to confer the phenotype in *T. b. brucei* [20]. Efforts to delete the gene from *T. b. gambiense* were unsuccessful and the function of TgsGP remained unknown [20]. Here we have successfully deleted the *TgsGP* gene from *T. b. gambiense* and demonstrated that it is essential for human serum resistance and requires a *T. b. gambiense* genetic background in order to function.

## Results

### Deletion of *TgsGP* in wild-type group 1 *T. b. gambiense*

**Figure 1:**
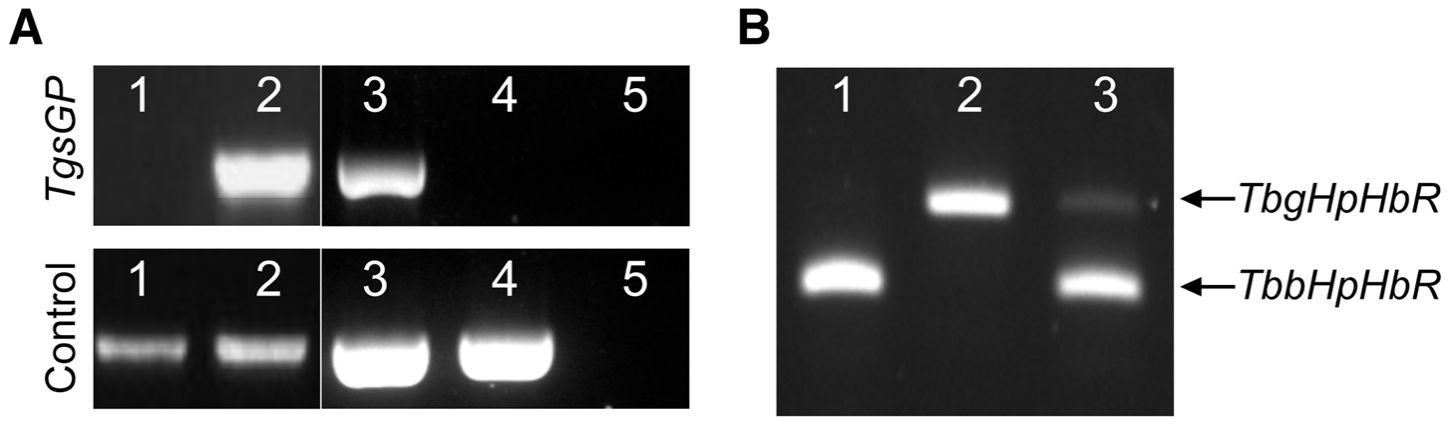
PCR amplification of *TgsGP* and RT-PCR of *HpHbR* in wild-type and transfected lines. (**A**) Amplification of *TgsGP* and a control gene (cathepsin L) by PCR in [1] wild-type *T. b. brucei* [2], *TgsGP* ^−/+^ *T. b. brucei* [3], wild-type *T. b. gambiense*, [4] *TgsGP* ^−/0^ *T. b. gambiense* [5] and negative control. (**B**) RT-PCR amplification of *HpHbR* followed by *Hpy*CH4V restriction digestion of [1] wild-type *T. b. brucei*, [2] wild-type *T. b. gambiense* and [3] *TbbHbHpR* ^−/+^ *TgsGP* ^−/0^ *T. b. gambiense*.

**Figure 2:**
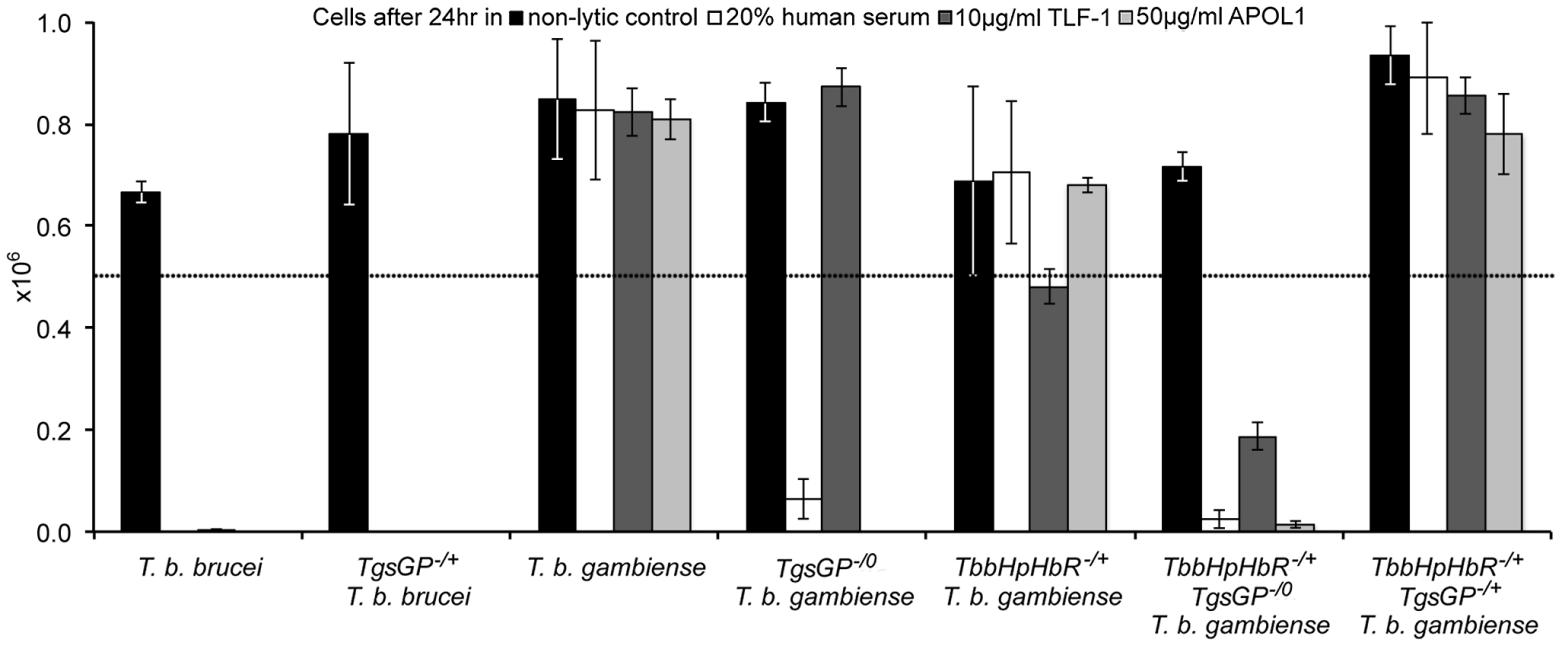
TgsGP is essential for resistance to human serum in *T. b. gambiense.* The number of surviving cells after 24% human serum (open box), 10 µg/ml TLF-1 (dark grey box), 50 µg/ml recombinant APOL1 (light grey box) or a non-lytic 20% FBS control (black box). The dotted line indicates the starting concentration of 5×10^5^ cells. The cell lines assayed were wild-type *T. b. brucei*; *TgsGP* ^−/+^ *T. b. brucei*; wild-type *T. b. gambiense*; *TgsGP* ^−/0^ *T. b. gambiense*; *TbbHbHpR* ^−/+^ *T. b. gambiense*; *TbbHbHpR* ^−/+^ *TgsGP* ^−/0^ *T. b. gambiense* and *TbbHbHpR* ^−/+^ *TgsGP* ^−/+^ *T. b. gambiense*. Standard error is shown, n = 4 for each data point.

To assess whether *TgsGP* is involved in human serum resistance in *T. b. gambiense*, the gene was deleted from the genome of a group 1 *T. b. gambiense* strain. All strains of *T. b. gambiense* investigated so far are hemizygous for *TgsGP*, allowing a complete knockout with just one round of transfection [20]–[22]. Although it was postulated that *TgsGP* was an essential gene and could not be deleted [20], several *TgsGP* ^−/0^ clones were generated in this study. One of the clones was selected for analysis and used for subsequent assays. The deletion of *TgsGP* from the clone was confirmed by PCR (Figure 1A). The *TgsGP* ^−/0^ *T. b. gambiense* clones was unable to survive in the presence of normal human serum (Figure 2) or recombinant APOL1 (Figure 2), with significantly fewer surviving cells compared to wild-type *T. b. gambiense* (human serum t-test p = 0.001, APOL1 t-test p<0.001). The clone grew in the presence of non-lytic serum in a similar manner to wild-type *T. b. gambiense* (t-test p = 0.145). This indicates that TgsGP is involved in protecting against the trypanolytic protein APOL1.

The clone was able to grow in the presence of TLF-1 and the number of cells after 24 hours does not differ significantly from that of the wild-type *T. b. gambiense* strain (t-test p = 0.511). Wild-type *T. b. gambiense* is resistant to lysis by TLF-1 due to reduced efficacy of their HpHbR for binding TLF-1. Thus lethal amounts of the lytic particle are not internalised by the parasites [18], [19]. It is likely that *TgsGP* ^−/0^ *T. b. gambiense* clones are able to grow in the presence of TLF-1 because it possesses the *T. b. gambiense HpHbR* allele that is less efficient at binding TLF-1.

### Deletion and reintroduction of *TgsGP* in *TbbHpHbR* ^−/+^ *T. b. gambiense*

**Figure 3:**
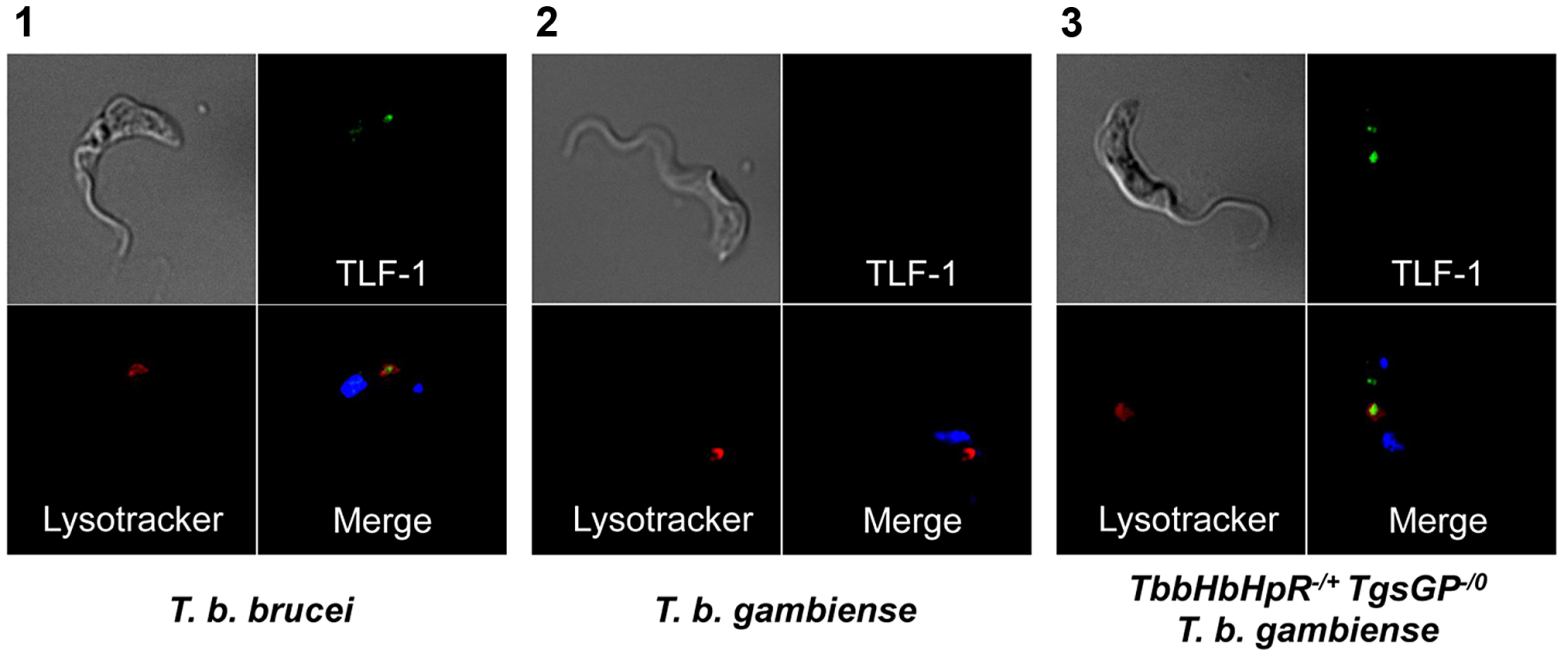
Uptake of TLF-1 across strains. Uptake of TLF-1 after one hour in [1] wild-type *T. b. brucei* [2] wild-type *T. b. gambiense* and [3] *TbbHbHpR* ^−/+^ *TgsGP* ^−/0^ *T. b. gambiense* by co-localization of fluorescently tagged TLF-1 (green) with the lysosomal marker Lysotracker (red). The kinetoplast and nucleus were also stained using DAPI (blue).

As previously detailed, group 1 *T. b. gambiense* is characterised by a non-functional HpHbR which results in a reduced uptake of TLF-1 and to a lesser extent TLF-2 [17]–[19], [24]. To investigate the effect of the loss of *TgsGP* in combination with TLF-1 uptake, a *T. b. gambiense* strain expressing a functional *T. b. brucei* HpHbR (*TbbHpHbR*) and lacking *TgsGP* was created (termed *TbbHpHbR* ^−/+^ *TgsGP* ^−/0^). Expression of both wild-type and ectopic *TbbHpHbR* alleles was confirmed by RT-PCR (Figure 1B). An allele-specific *Hpy*Ch4V restriction site present in the open reading frame of *TbbHpHbR*, but absent in *TbgHpHbR*, was used to distinguish between the alleles (Figure 1B) and demonstrated that both alleles were expressed, although the *TbgHpHbR* allele exhibits lower expression relative to the *TbbHpHbR* allele. The strain expresses a fully functional HpHbR and hence takes up TLF-1 to a degree similar to *T. b. brucei*, confirmed by fluorescence microscopy (Figure 3). *TbbHpHbR* ^−/+^ *TgsGP* ^−/0^ *T. b. gambiense* clones were killed in the presence of normal human serum, recombinant APOL1 or, unlike *TgsGP* ^−/0^ clones, physiological levels of TLF-1 (Figure 2). The number of remaining cells at 24 hours was significantly lower than wild-type *T. b. gambiense* (human serum t-test p = 0.001, TLF-1 t-test p<0.001, APOL1 t-test p<0.001). However, the cells were able to grow in the presence of non-lytic serum in a similar manner to wild-type *T. b. gambiense* (t-test = 0.690). A *T. b. gambiense* clone with TgsGP and the functional *Tbb*HpHbR was able to grow in the presence of human serum and APOL1 (Figure 2) with cell number not significantly differing from wild-type *T. b. gambiense* (human serum t-test p = 0.936, APOL1 t-test p = 0.465) or in the presence of non-lytic serum (t-test p = 0.972). However, the clone displayed a trypanostatic growth effect in physiological levels of purified TLF-1 with significantly fewer surviving cells compared to wild type (Figure 2) (t-test p = 0.001).

To confirm that the loss of resistance to human serum, APOL1 and TLF-1 in *TbbHpHbR* ^−/+^ *TgsGP* ^−/0^ *T. b. gambiense* was due to the loss of *TgsGP*, the gene was re-introduced into this background. Resistance to human serum, TLF-1 and APOL1 was rescued by the re-introduction of *TgsGP*, confirming that this gene is essential for resistance to lysis (Figure 2). When the same *TgsGP* add-back construct was transfected into a human serum sensitive *T. b. brucei*, it did not confer resistance to any lytic component (Figure 2), confirming earlier work [20].

### Localisation of TgsGP

**Figure 4:**
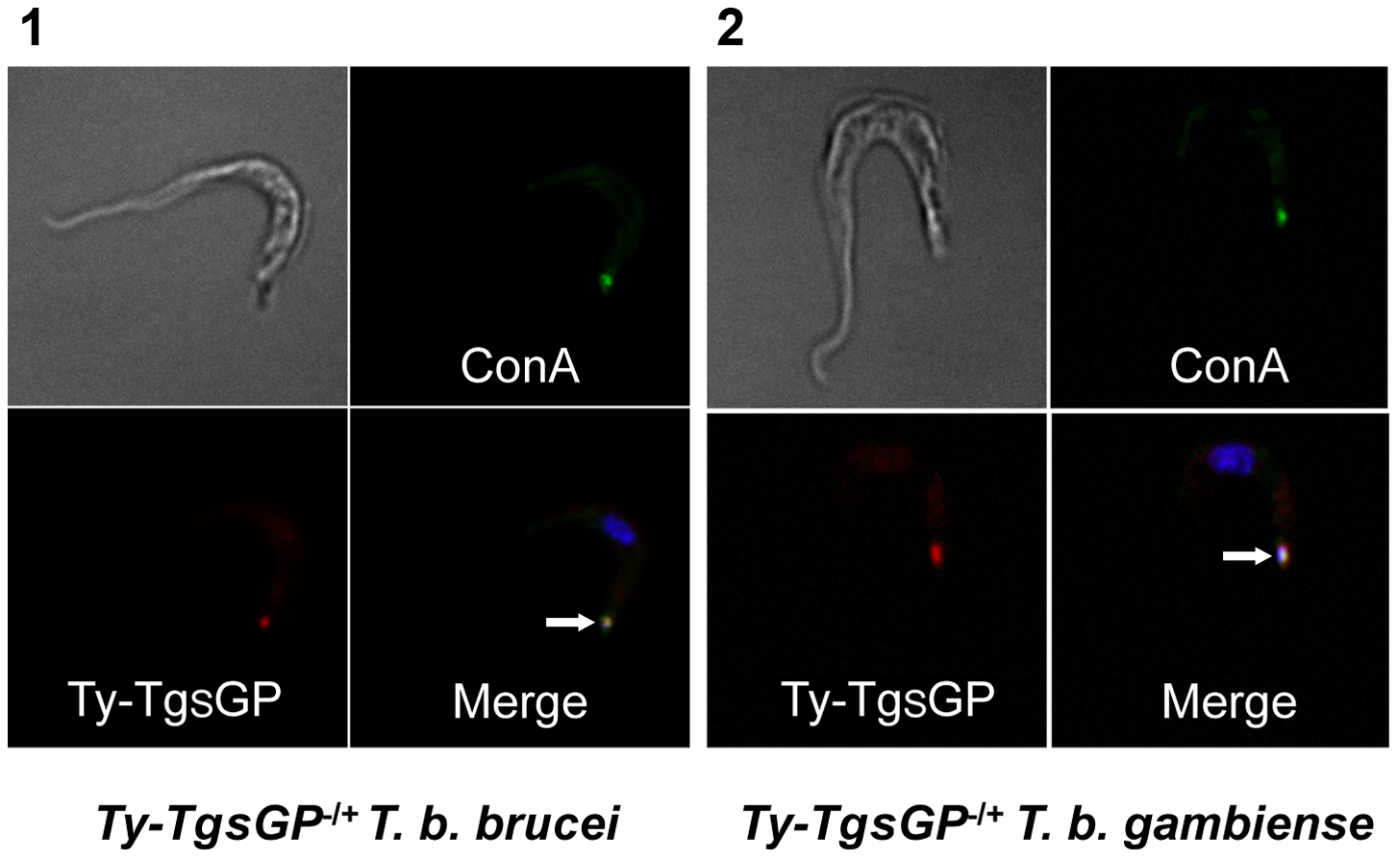
Localisation of TY-TgsGP. Localisation of TY-tagged TgsGP (red) relative to un-endocytosed FITC-labeled Concanavalin A bound to glycoproteins in the flagellar pocket (green) and DAPI stained nucleus and kinetoplast (blue) in [1] TY-*TgsGP* ^−/+^ *T. b. brucei* and [2] *TbbHbHpR* ^−/+^ TY-*TgsGP* ^−/+^ *T. b. gambiense*. The flagellar pocket (revealed by Concanavalin A and kinetoplast position) is indicated with a white arrow.

Previous work has shown that TgsGP localises to the flagellar pocket in *T. b. gambiense* and this is likely to be the site of interaction between TLF and TgsGP [20]. A possible hypothesis for the observation that when TgsGP is transfected into in *TbbHpHbR* ^−/+^ *TgsGP* ^−/0^ *T. b. gambiense* background it restores human serum resistance but does not confer resistance in *T. b. brucei* [20] (figure 2) is that the protein is not trafficked correctly to the flagellar pocket. In order to verify localisation, *TgsGP* was transfected into wild-type *T. b. brucei* with the addition of a TY tag into a *Hin*dIII restriction site at position 1130 of the *TgsGP* ORF, upstream of the predicted GPI anchor sequence [25], [26]. Immunofluorescence with anti-TY antibodies shows clear localisation of TY-TgsGP adjacent to the kinetoplast and co-localization with fluorescent Concanavalin A, which acts as a marker for the flagellar pocket [27], (Figure 4). However, these cells were killed in human serum, TLF-1 or APOL1 (Figure S1). A similar localisation is observed when the TY-tagged TgsGP protein is expressed in *TbbHpHbR* ^−/+^ *TgsGP* ^−/0^ *T. b. gambiense* (Figure 4), with strong signal close to the kinetoplast and a more diffuse signal closer to the nucleus. In this case, the capacity to grow in human serum, TLF-1 and APOL1 was restored by the reintroduction of the TY-tagged *TgsGP* (Figure S1). As an identical construct was used in both transfections, it is probable that group 1 *T. b. gambiense* possess a protein or mechanism complementing TgsGP that is absent in *T. b. brucei*.

## Discussion

This study demonstrates that the *TgsGP* gene is essential for resistance to human serum in the most clinically important *T. brucei* sub-species, group 1 *T. b. gambiense*. Previous work has shown that *TgsGP* did not confer resistance to human serum when ectopically expressed in *T. b. brucei* [20], which was confirmed here. As originally hypothesized [20], it appears likely that this is due to other factor(s) or mechanism(s) that works in concert with TgsGP, which are absent in *T. b. brucei*. By removing *TgsGP* from *T. b. gambiense* itself, we have demonstrated that the gene is necessary for resistance to human serum. Elucidation of a gene essential to human serum resistance in group 1 *T. b gambiense* unlocks new avenues for future treatment of human African sleeping sickness. These include peptide screens that neutralise the TgsGP protein, targeted antibodies or the possibility of using TgsGP as a vaccine candidate, as expression is required for parasite survival in humans. Additionally, there exists the potential that variants of APOL1 may offer protection against *T. b. gambiense*. Sera from individuals possessing certain APOL1 alleles has been shown to affect the growth of *T. b. rhodesiense* and it has been suggested that these alleles may be protective against *T. b. rhodesiense* [28], [29]. However, this has yet to be confirmed in a case control study. Nevertheless, it is likely that there are variant APOL1 alleles that protect against group 1 *T. b. gambiense* in resistant individuals, such as the reportedly resistant Bambuti people of the Mbomo region in the Democratic Republic of the Congo [30] or recently described asymptomatic and self-cured cases from Côte d'Ivoire [31].

One other benefit of our study is the trypanosome research community now possesses a representative group 1 *T. b. gambiense* strain that is easily cultured, is no longer human serum resistant, yet only differs from the wild-type by a single gene. This is a powerful biological resource that could replace *T. b. brucei* as the common laboratory model for the human disease, which maybe useful, particularly as several drugs display different efficacies between sub-species [1]. As such, identifying *TgsGP* as a gene essential for resistance to human serum in group 1 *T. b. gambiense* will likely be important to future control of the disease.

## Materials and Methods

### Trypanosomes strains and maintenance

Bloodstream form *T. b. brucei* Lister 427 (MITat 1.2) was grown at 37°C under 5% CO_2_ in HMI9 medium supplemented with 20% foetal bovine serum (Sigma-Aldrich) and 20% Serum-Plus (Sigma-Aldrich). The bloodstream form group 1 *T. b. gambiense* strain ELIANE (MHOM/CI/52/ELIANE) was isolated from a patient infected while in Côte d'Ivoire [22]. It was cultured in modified HMI9 [32] supplemented with 20% serum plus (SAFC Biosciences Ltd.). Similar to other group 1 *T. b. gambiense* strains, ELIANE is consistently resistant to lysis by human serum, despite repeated passage.

### Transfection of *T. b. brucei* and group 1 *T. b. gambiense*

*T. b. gambiense* and *T. brucei* strains were transfected using the protocols outlined in [33]. For ectopic expression of *TgsGP* in *T. b. brucei* and reinsertion into the *TgsGP* ^−/0^ *T. b. gambiense* strains, the *TgsGP* ORF was inserted into the pURAN vector [34] using G418 for selection. Ectopic expression of *TbbHbHpR* in *T. b. gambiense* was achieved using the tubulin-targeting *TbbHbHpR* pTub-phelo construct, using phleomycin for selection [17]. For deletion of *TgsGP* from the genome of *T. b. gambiense* and *TbbHbHpR* ^−/+^ *T. b. gambiense*, 500 base pairs from both the upstream and downstream regions of *TgsGP* (sequence AM237444.1, http://www.genedb.org) were inserted into a vector containing a hygromycin resistance cassette. Insertion of TY-tagged *TgsGP* into the deletion strain *T. b. gambiense* and *T. b. brucei* was performed by inserting a TY tag into a *Hin*dIII restriction site at position 1130 of the *TgsGP* ORF. This sequence was ligated into the pURAN vector [34], [35] and transfectants were screened using a G418 selection marker. This insertion site is upstream of the predicted GPI anchor site identified using the big-PI software package [25] and a GPI prediction protocol validated for trypanosomes [26]. Correct integration for constructs was assessed by PCR and/or RT-PCR. All primers used in the studies and their targets, are listed in Table S1.

### RT-PCR of expressed *Tbb*HpHbR in group 1 *T. b. gambiense*

Total RNA was isolated from cells using RNeasy kit (Qiagen) according to manufacturers' instruction, with additional DNase steps. 2 µg RNA was subject to a second round of DNase treatment (Invitrogen) prior to cDNA synthesis using Superscript III (Invitrogen), according to manufacturers' instructions. RT-PCR was performed using *Taq* DNA polymerase and the primers are described in Table S1. For RFLP analysis of *HpHpR*, the amplified product was cleaned using GeneJet PCR purification column, digested with *Hpy*Ch4V and the digested products separated on a 2% agarose gel.

### TLF-1 purification

TLF-1 purification, labeling and survival assays were performed as previously described [17], [36].

### Generation of recombinant APOL1

APOL1 synthesis and purification was performed as previously described [36]. Protein purity was estimated using a Nanodrop spectrometer (Nanodrop) and SDS-PAGE. A Western blot using an antibody raised against an APOL1 peptide (Sigma-Aldrich) was used to verify that the bands present were APOL1.

### Lysis survival assays

To assess survival in human serum, trypanosomes were diluted to 5×10^5^ per ml in HMI9 and incubated for 24 hours with 20% human serum or 20% non-lytic foetal bovine serum (FBS). The number of surviving trypanosomes in each well was recorded after 24 hours using a haemocytometer. To assess survival in TLF-1 and APOL1, trypanosomes were diluted to 5×10^5^ per ml in HMI9 with FBS. Cells were incubated with a physiological amount of TLF-1 (10 µg/ml). For the recombinant APOL1 assays, a concentration of 50 µg^−1^ ml was used as this had previously been determined to kill 100% of *T. b. brucei* cells in a 24-hour assay [36]. The number of cells in each well was counted with a haemocytometer at 24 hours. There were four replicates for each data point. The number of surviving cells for each treatment were compared between each of the *T. b. gambiense* clones and wild-type *T. b. gambiense* using the unpaired 2-tailed t-test function of the Minitab 14 Statistics Package (Minitab).

### Immunofluorescence assays

TLF-1 immunofluorescence assays were performed as previously described [17], [36]. Immunofluorescence localisation of TY-TgsGP was performed with approximately 10^6^ bloodstream-cultured parasites in mid-log phase. Cells were incubated with 5 mg/ml FITC conjugated Concanavalin A in serum-free HMI9 for 20 minutes at 4°C. The Concanavalin A binds to glycoproteins in the flagellar pocket but is not endocytosed due to the reduced temperature, thus labeling the flagellar pocket [27]. Cells were then fixed by immersion in chilled methanol for 30 minutes. Slides were incubated for 1 hour with 1∶500 primary mouse anti-TY antibody (Iain Johnston, University of Glasgow), washed with PBS and then incubated with 1∶1000 of AlexaFluor568 anti-mouse secondary (Invitrogen). The slides were mounted using 50% glycerol, 0.1% DAPI and 2.5% DABCO. Parasites were imaged using a Deltavision Core system and SoftWorx package (Applied Precision). Images were composited using the ImageJ software package [37].

## Supporting Information

Figure S1 **TY-TgsGP behaves similarly to TgsGP.** The number of surviving cells after 24 hours incubation with 20% human serum (open box), 10 µg/ml TLF-1 (dark grey box), 50 µg/ml recombinant APOL1 (light grey box) or a non-lytic 20% FBS control (black box). The dotted line indicates the starting concentration of 5×10^5^ cells. The cell lines assayed were TY-*TgsGP* ^−/+^ *T. b. brucei* and *TbbHbHpR* ^−/+^ TY-*TgsGP* ^−/0^ *T. b. gambiense*. Standard error is shown, n = 2 for each data point.
(DOCX)

Table S1 **Primers used and their function.**
(DOCX)
