# Supplementary figures and images for: The TgsGP Gene Is Essential for Resistance to Human Serum in Trypanosoma brucei gambiense
Source: PLoS Pathog. 2013 Oct 3;9(10):e1003686. doi: 10.1371/journal.ppat.1003686 (PMC3789759; doi:10.1371/journal.ppat.1003686)

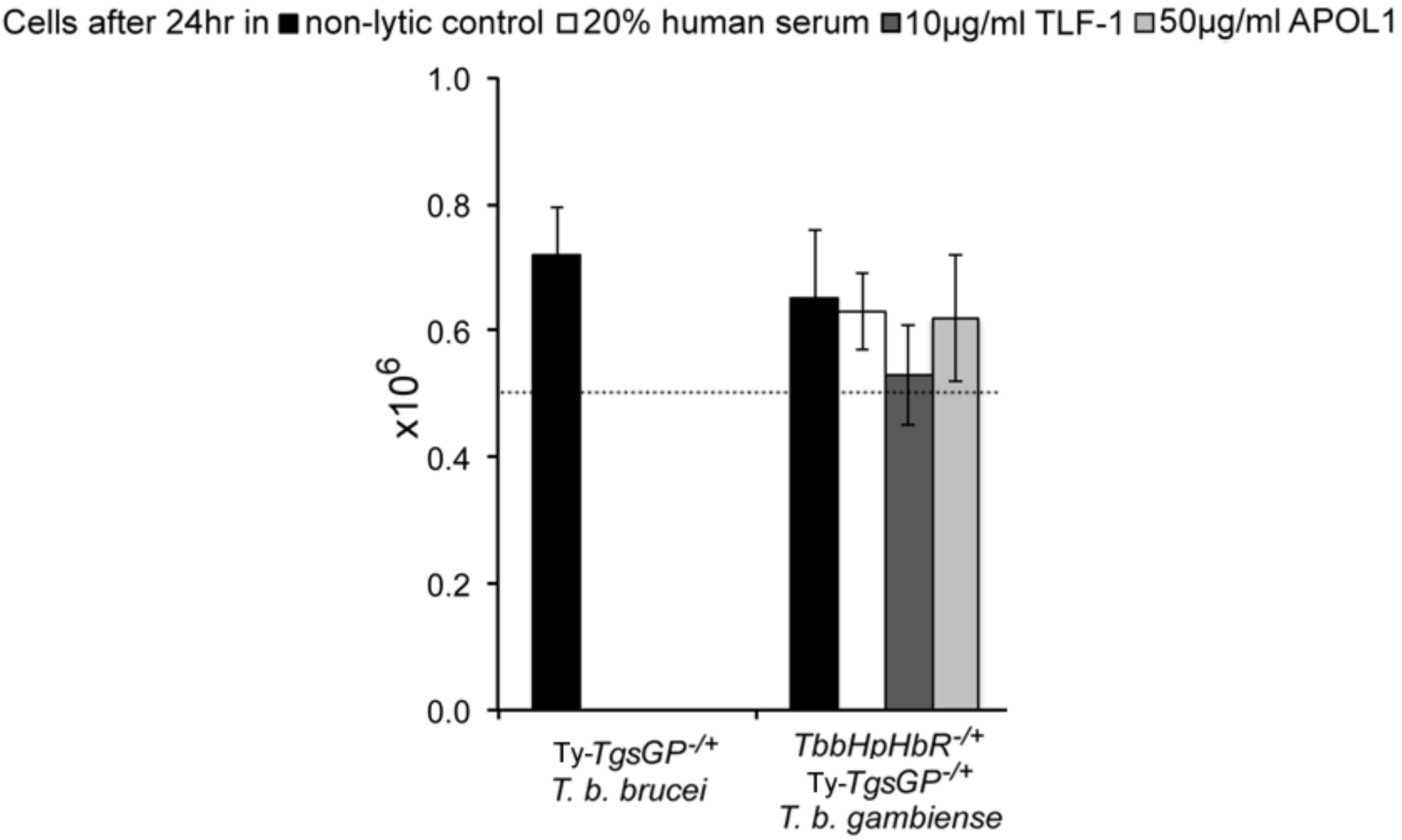

Supplement: Figure S1 — TY-TgsGP behaves similarly to TgsGP. The number of surviving cells after 24 hours incubation with 20% human serum (open box), 10 µg/ml TLF-1 (dark grey box), 50 µg/ml recombinant APOL1 (light grey box) or a non-lytic 20% FBS control (black box). The dotted line indicates the starting concentration of 5×10^5^ cells. The cell lines assayed were TY-TgsGP −/+ T. b. brucei and TbbHbHpR −/+ TY-TgsGP −/0 T. b. gambiense. Standard error is shown, n = 2 for each data point. (DOCX) [file ppat.1003686.s001.docx]
