# Supplementary material for: The TgsGP Gene Is Essential for Resistance to Human Serum in Trypanosoma brucei gambiense
Source: PLoS Pathog. 2013 Oct 3;9(10):e1003686. doi: 10.1371/journal.ppat.1003686 (PMC3789759; doi:10.1371/journal.ppat.1003686)
Supplement: Table S1 — Primers used and their function. (DOCX) [file ppat.1003686.s002.docx]

Supplementary 1. Primers used and their function.

| **Oligo** | **Gene** | **Sequence 5’-3’** | **Notes/Use** |
| --- | --- | --- | --- |
| 35-GAPDH | *GAPDH* | TCACAGTGAAATCTGCTGCC | RT-PCR control |
| 36-GAPDH | *GAPDH* | TCGCAATGAAGGTAAGGTCC | RT-PCR control |
| 121-HygF | hygromycin B phosphotransferase | ATGAAAAAGCCTGAACTCAC | Hyg Integration |
| 125HygR2 | hygromycin B phosphotransferase | GCTCCATACAAGCCAACCAC | Hyg Integration |
| 126HygR3 | hygromycin B phosphotransferase | CTATTCCTTTGCCCTCGGACG | Hyg Integration |
| 224-CatLF | Cathepsin L-like  Cysteine peptidase | CAGTGACCCCAGTGAAGGAT | PCR control |
| 225-CatLR | Cathepsin L-like  Cysteine peptidase | GAGACATTGGTTTGTGCCCT | PCR control |
| 230-TgsGF | *TgsGP* | CGTCAGCAGCAAAGGTGTTA | PCR |
| 231-TgsGPR | *TgsGP* | CACATCTGCCAGTGTCTGGT | PCR |
| 268_TgGP+EcoRVF | *TgsGP* | CATGGATATCATGTGGCAATTACTAGCAAT | Cloning *TgsGP* into pRM482 |
| 264_TgGP_5FlnkF1 | *TgsGP* 5’ flank | GATGGGCATCATACACAAGG | *TgsGP* KO 5’ integration |
| 269_TgGP+EcoRVR | *TgsGP* | CATGGATATCGTGTTAAAATATATCAAATAGCAAAGTTC | Cloning *TgsGP* into pRM482 |
| 327_5'tub | *Tubulin* 5’UTR | GAGCTAGTGAGATCAACAGTAC | Tubulin 5’ integration |
| 328_3'tub | *Tubulin* 3’UTR | AGCCTGAACGCAGACGATTTC | Tubulin 3’ integration |
| 6.440.seq3R | *HpHbR* | CGCTTCATCTCTTCCTGTGC | RT-PCR *HpHbR;* Integration of *HpHbR* into tubulin*,* RFLP analysis |
| RealT.6.440B_F | *HpHbR* | ACCTCCGCCAGAGAAAATCTC | RT-PCR *HpHbR;* Integration of *HpHbR* into tubulin, RFLP analysis |
| TgsGP outerF1 | *TgsGP* | TCACGGCCATCAGACGGAGA | PCR and RT-PCR |
| TgsGP Anti | *TgsGP* | GCCATCGTGCTTGCCGCTC | PCR and RT-PCR |
| Oligo dT | NA | GCTCTAGATTTTTTTTTTTTTTTTTTTT | Target 3’ PolyA tail to sequence transcript |
| Splice leader RNA | NA | CCGGAATTCGCTATTATTAGAACAGTTTCT | Target 5’ Splice leader to sequence transcript |
| TgsGP pURAN_F | *TgsGP* | GATCGGGCCCGAATTCATGTGGCAATTACTAGCAAT | Cloning *TgsGP* into pURAN |
| TgsGP pURAN_R | *TgsGP* | GATCGCGGCCGCGAATTCTTAAAAAAGCAAAAATGCAAGC | Cloning *TgsGP* into pURAN |
| QC Ava TgsGP pURAN_F | *TgsGP* | CAATTGAGGCAGGAGCACGAGCGGCAAGCACGATGGC | Verification of *TgsGP* in pURAN |
| QC Ava TgsGP pURAN_R | *TgsGP* | GCCATCGTGCTTGCCGCTCGTGCTCCTGCCTCAATTG | Verification of *TgsGP* in pURAN |
| RT TgsGP | *TgsGP* | CGTAGCTTACTGCGGTTACG | Quantifying expression of *TgsGP* using splice leader |
| RT HpHbR | *HpHbR* | GATCGAATTCCTACACCACCACCTGGAGCA | Quantifying expression of  *HpHbR* using splice leader |
| RT HpHbR nested | *HpHbR* | TTAGACAATTTAAACTTGTTCAGC | Quantifying expression of *HpHbR* using splice leader |
| RT enolase | Enolase | CCAACCGGGAAAGCCAAATTTAGC | Quantifying expression of the Enolase control using splice leader |
